# Case report: Paracorporeal lung assist device for 215 days as a bridge-to-lung transplantation in a patient with bronchopulmonary dysplasia and severe pulmonary hypertension

**DOI:** 10.3389/frtra.2023.1197906

**Published:** 2023-07-06

**Authors:** Sebastian G. Michel, Maja Hanuna, Joseph Pattathu, Jelena Pabst von Ohain, Christian Schneider, Theresa Kauke, Nikolaus Kneidinger, Juergen Behr, Katrin Milger, Juergen Barton, Tobias Veit, Christine Kamla, Christoph Mueller, Robert Dzieciol, Lauren Christen, Michael Irlbeck, Roland Tomasi, Jan Abicht, Patrick Scheiermann, Matthias Feuerecker, Robert Dalla-Pozza, Marcus Fischer, Andre Jakob, Matthias Hermann, Nikolaus Haas, Christian Hagl, Jürgen Hörer

**Affiliations:** ^1^ Division of Congenital and Pediatric Heart Surgery, Department of Cardiac Surgery, Ludwig Maximilian University Munich, Munich, Germany; ^2^ Department of Congenital and Pediatric Heart Surgery, German Heart Center Munich, Technical University of Munich, Munich, Germany; ^3^ Comprehensive Pneumology Center Munich, German Center for Lung Research (DZL), Munich, Germany; ^4^ Department of Cardiac Surgery, Ludwig Maximilian University Munich, Munich, Germany; ^5^ Department of Pediatric Cardiology and Intensive Care, Ludwig Maximilian University Munich, Munich, Germany; ^6^ Department of Thoracic Surgery, Ludwig Maximilian University Munich, Munich, Germany; ^7^ Department of Medicine V, Pulmonology, Ludwig Maximilian University Munich, Munich, Germany; ^8^ Department of Anesthesiology, Ludwig Maximilian University Munich, Munich, Germany; ^9^ Munich Heart Alliance, German Center for Cardiovascular Research (DZHK), Munich, Germany

**Keywords:** lung assist device, lung transplantation, pulmonary hypertension, bridge-to-lung transplant, ECMOextracorporeal membrane oxygenation

## Abstract

Pulmonary hypertension (PH) is a known and life limiting complication of preterm born young adults with bronchopulmonary dysplasia (BPD), ultimately leading to progressive right ventricular (RV) failure. Prognosis remains poor, especially in patients unresponsive to modern vasoactive pharmacotherapy. Therefore, lung transplantation presents the treatment of choice to avert cardiac failure. With limited donor organ availability and long waiting times, the implantation of a paracorporeal lung assist device (PLAD) is a way to bridge the patient as an alternative to veno-arterial ECMO. Herein, we present the case of a prematurely born 23-year-old female, who developed severe PH due to BPD and consequently experienced therapy refractory RV failure. Urgent PLAD implantation was performed and the patient successfully underwent double-lung transplantation after 215 days of PLAD support. No major PLAD-associated complications occurred and full recovery of RV function could be observed after double-lung transplantation.

## 1. Introduction

Bronchopulmonary dysplasia (BPD) is a consequence of abnormal bronchopulmonary development in preterm born infants, especially if born prior to a gestational age of 28 weeks (1, 2). Its association with pulmonary vascular disease in terms of dysmorphic capillary configuration and abnormal vascular remodeling poses an increased risk for pulmonary hypertension (PH) and approximately 20%–44% of preterm born infants with BPD are affected (1, 3–6). Whereas advances in ventilation and vasoactive treatment strategies for infants with BPD led to improved outcome, survivors remain at risk for developing PH and consequently right ventricular (RV) failure, even in adulthood (5–7). Therefore, further screening of patients with BPD for PH has been recommended in recently published guidelines (8). The initial treatment of PH consists of vasoactive agent therapy, and lung transplantation is preferred in selected patients unresponsive to optimal medical treatment. However, with limited donor organ availability, waiting times often exceed survival. Paracorporeal lung assist devices (PLAD) have been introduced as a possible bridge-to-transplantation, however evidence regarding outcome is still insufficient (9, 10). We report on a 23-year-old female patient, who had been listed for lung transplantation due to severe PH. She deteriorated and went into RV failure, so emergency PLAD implantation was performed as a bridging therapy. She successfully underwent lung transplantation after 215 days of PLAD support.

## 2. Case description

We describe the clinical course of a 23-year-old female, who was prematurely born at gestational week 27. A ventriculoperitoneal shunt was placed for hydrocephalus relief. She suffered from bronchopulmonary dysplasia and ultimately developed severe PH.

While cognitive development was normal, physical growth remained impaired (height 142 cm and weight 34 kg). At age 23, she presented with a progressive decline in exertional capacity and significant dyspnea (NYHA III-IV). Clinical examination showed central cyanosis, severe peripheral edema, clubbing of nails, signs of hepatomegaly and mild hypoxemia with continuous oxygen demand (2 L/min) to maintain saturations above 90%. Laboratory findings included an increased NT-proBNP (3,546 pg/ml) but normal levels of kidney and liver parameters. ECG depicted right axis deviation and signs of RV-hypertrophy. Echocardiography revealed a dilated right atrium and reduced RV function (tricuspid annular plane excursion: 12 mm), severe tricuspid valve regurgitation, congestion of the hepatic veins and signs of severe pulmonary hypertension (D-sign and a systolic pulmonary pressure: 83 mmHg + central venous pressure at systemic arterial pressures of 119/65 mmHg). Left ventricular ejection fraction was within normal range (Figure 2A). After recompensation with forced diuresis, right heart catheterization showed the following results: mean pulmonary pressure 60 mmHg, left atrial pressure 6 mmHg, transpulmonary pressure gradient 54 mmHg, pulmonary vascular resistance 24 Wood units, right atrial pressure 10 mmHg and a cardiac index of 1.9 L/min/m^2^. Arterial oxygen saturation at room air was 87%. According to the Sitbon criteria (11), pulmonary hypertension was not reversible with vasoactive agents (oxygen, nitric oxide and ilomedin were applied). The patient deteriorated despite treatment with Riociguat, Macitentan, oral prostacyclin and intravenous administration of Treprostinil via a subcutaneously implanted pump. In the following months, the patient suffered from frequent pulmonary infections with intermittent oxygen demand and a second cardiac decompensation occurred. In the mean time, the patient had been listed for double lung transplantation, however, her clinical status quickly deteriorated in terms of increased oxygen demand and right ventricular failure (NT-proBNP = 11,485 pg/ml) due to now suprasystemic pulmonary pressures (systolic pulmonary pressure 120 mmHg, central venous pressure 26 mmHg). As the waiting time for lung transplantation in Germany remains long for patients smaller in size, the interdisciplinary decision for urgent PLAD implantation as a bridge-to-transplantation was made. Preoperatively, a loading dose of Levosimendan was administered.

**Figure 1:**
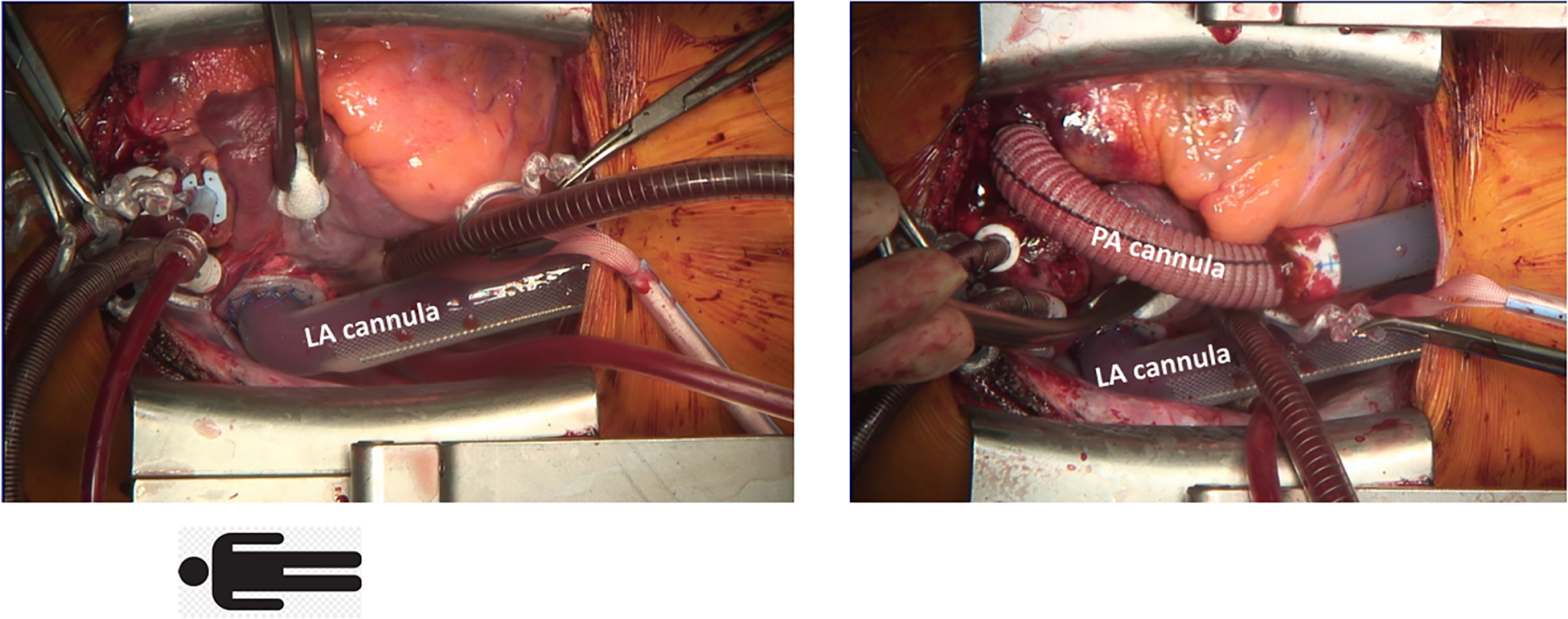
Implantation of a paracorporeal lung assist device, cannulation of the left atrium (LA) and main pulmonary artery (PA).

**Figure 2:**
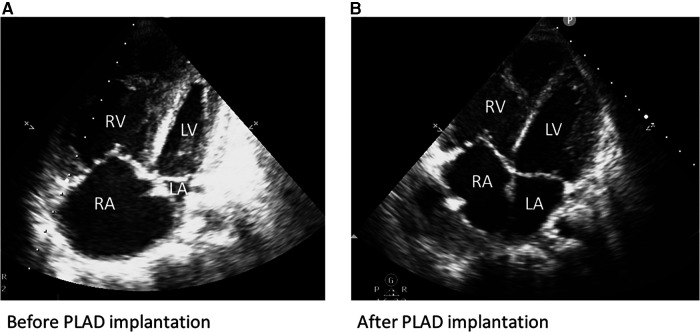
(**A**) Transthoracic echocardiography before PLAD implantation showing signs of RV failure. (**B**) Transthoracic echocardiography after PLAD implantation: recovery of RV function and LV volume conditioning.

**Figure 3:**
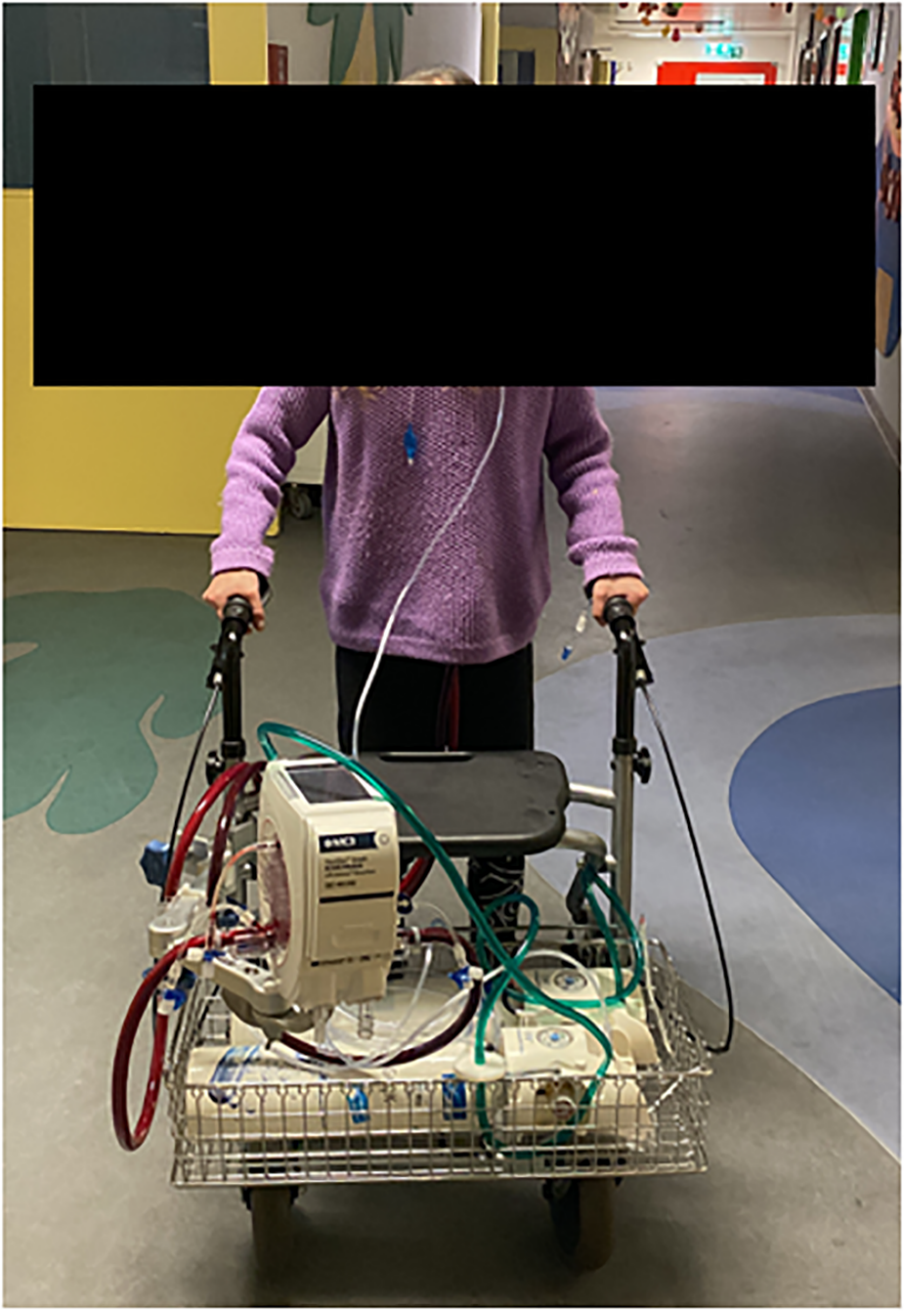
Physical therapy while on PLAD.

After careful induction of general anesthesia, rapid median sternotomy was performed, and cardiopulmonary bypass was established through bicaval and aortic cannulation. An atrial Berlin Heart EXCOR® cannula (9 mm) was inserted into the left atrium with 8 interrupted sutures (Prolene 4-0) enhanced with felt pledgets. A Berlin Heart EXCOR® graft adapter cannula (9 mm), extended by 12 mm Gelweave Vascutec Terumo straight graft prosthesis was sewn to the pulmonary artery with a continuous Prolene suture (Figure 1). Both cannulae were channeled subcutaneously to the subxiphoid area, de-aired and connected to a Medtronic Nautilus™ oxygenator with an incorporated heat exchanger. This ultimately resulted in a passive shunt from the pulmonary artery (PA) to the left atrium (LA) with a flow of 2.5 L/min, and prompt hemodynamic stabilization could be achieved. Weaning from cardiopulmonary bypass was uneventful, and the chest was closed primarily. The pressure gradient across the oxygenator was 12 mmHg. Postoperatively, the patient required renal replacement therapy for 3 weeks due to acute renal failure and a tracheostomy was performed two weeks post PLAD implantation. As pulmonary pressures decreased (systolic pulmonary pressure approximately 70 mmHg; 2/3 of systemic systolic pressure), Treprostinil was discontinued, right ventricular function progressively improved and inotropic support could be discontinued (Figure 2B). The anticoagulation regime included Aspirine, Clopidogrel and Vitamin K antagonists (target INR 3–3.5). Nurses and perfusionists checked the whole system 3 times a day. When clot formation >2 mm was visible, the system was changed at the bedside with the patient being awake. The patient required more than 20 exchanges of the tubing system or solely of the oxygenator due to frequent thrombus formation. Systemic embolization could be successfully averted with frequent PLAD inspections and liberal system exchanges. No major complications occurred during PLAD support, and the patient recovered to the point of being able to walk on the hospital ward (Figure 3). The patient underwent lung transplantation after 215 days of PLAD support. No thrombotic material was found in the Berlin Heart EXCOR® cannulae or the left atrium. After lung transplant, the patient was put on central veno-arterial ECLS support for prolonged weaning, which is a standard procedure for patients with severe PH at our institution and the chest remained open. A single rethoracotomy was performed on the first day post-transplant due to bleeding and the patient underwent ECLS-explantation and final sternal closure on the 5th postoperative day. She required three weeks of renal replacement therapy. The tracheostomy was closed six weeks after lung transplant. The patient was discharged in good clinical condition on the 54th day after lung transplant without supplementary oxygen and only slightly impaired renal function. Echocardiography revealed a remodeled right ventricle with adequate size as well as function and only minor tricuspid valve insufficiency.

### 2.1. Timeline


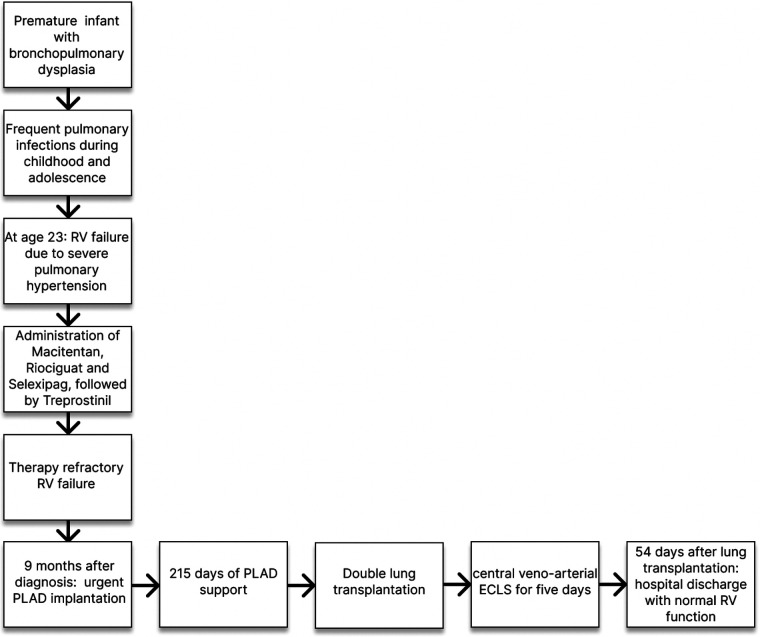


RV, right ventricle, PLAD, paracorporeal lung assist device, ECLS, extracorporeal life support.

## 3. Discussion

In patients with systemic pulmonary pressures, the creation of a PA-LA shunt results in a passive blood flow through the paracorporeal system containing a low resistance oxygenator, which is crucial to avoid the need of a pump as a driving force (12, 13). Avoiding pump-associated complications (e.g., hemolysis), this concept enables longer support times in comparison to veno-arterial extracorporeal membrane oxygenation. As our patient suffered from RV failure and faced an additionally extended waiting time for transplantation due to the low body surface area (1.18 m^2^), a long-term solution for cardiopulmonary support was required. Therefore, we decided to proceed with PLAD implantation instead of veno-arterial extracorporeal membrane oxygenation. Furthermore, PLAD leads to an increase in left ventricular preload, which preconditions the left ventricle and avoids LV failure after lung transplant (13). One requirement for successful PLAD implantation is preserved LV function (10). The option of intensified physical therapy after extubation and thus improving pre-transplant physical condition presents an additional advantage of PLAD over veno-arterial ECMO which always has peripheral cannulae in the bridge-to-transplant setting. Peripheral cannulation has a particularly high morbidity in pediatric and small patients (10).

The Medtronic Nautilus™ Smart ECMO Module oxygenator was chosen for three reasons: first, the design with a circular oxygenator profile and the transverse blood flow minimizes pressure drop, resulting in a low transoxygenator pressure gradient. In our patient, the pressure gradient remained <15 mmHg at flow rates of 2–2.5 L/min. Second, it contains a heat exchanger, which was necessary to counteract heat loss along the paracorporeal circuit. Third, continuous monitoring of pressure and oxygenation data via the oxygenator screen provides live information in terms of pulmonary pressures and RV unloading, which was particularly helpful in the early postoperative phase.

To our knowledge, this is the longest time of PLAD-support (>7 months) ever reported without PLAD-associated systemic embolic complications. Nevertheless, with a passive and consequently low blood flow along the paracorporeal circuit, patients remain at substantial risk for thromboembolic events. Therefore, we opted for an anticoagulant strategy including a target INR of 3–3.5 as well as dual antiplatelet therapy, mimicking the strategy after Berlin Heart ventricular assist device implantation for heart failure at our center. Nevertheless, we saw recurrent thrombus depositions, mostly at the connectors and to a lesser extent in the oxygenator without clinical relevance for the patient. We think the passive flow in this system (without a pump) is mainly responsible for the thrombogenicity. This underlines that technical improvement is necessary to reduce the risk of thromboembolic complications.

PLAD as a therapeutic approach has been introduced in 2009 and, to this day, reports are limited to a small number of patients with significantly shorter support times compared to our case (10, 12–16). De Perrot et al. described 4 cases of PA-LA Novalung implantations due to PH and RV failure (support time 9–69 days, cannulation of LA with right-angle cannula and PA with straight cannula) using unfractionated heparin (target activated clotting time 160–200 s) for anticoagulation and reported that none of the patients suffered from embolic complications (14). All of the patients underwent double lung transplantation and one patient died due to primary graft dysfunction. The authors concluded that an aggressive approach using circulatory support in patients with PH (VA ECMO and PA-LA Novalung) could effectively reduce waitlist mortality without increasing the risk for severe primary graft dysfunction (14). Strueber et al. presented the clinical course of four patients (support time 8–30 days) receiving the Novalung (PA-LA through median sternotomy or left thoracotomy) due to severe PH and RV failure (12). All patients survived transplantation, one patient underwent heart-lung transplantation and three patients received double lung transplantation. Since recovery of RV function and improved LV-filling and -function was always seen after PLAD, the authors concluded that this technique can avoid combined heart lung transplantation for the diagnosis of pulmonary hypertension.

While these results seem promising, pediatric patients tend to have worse outcome. Hoganson et al. reported on 4 pediatric patients (one neonate) who underwent PLAD implantation using the Novalung or Maquet Quadrox-iD oxygenator (support time 5–74 days) with a mortality rate of 50% and a stroke rate of 75%—possibly related to thrombus buildup in the left atrium and the left atrial metal tip cannula that was used in the first three cases (10). Thrombus formation (however without clinical relevance) was also seen at the right angle LA cannulae in de Perrot’s study (14). The Hoganson group therefore developed a novel technique of LA cannulation by creation of an ASD and suturing a Goretex graft (as an extension attached to the Berlin Heart cannula) into the rim of the ASD. The right atrium was closed around the Goretex tube. That way, there was no material in the LA that could cause thrombus formation.

In our patient, we implanted a Berlin Heart atrial cannula into the LA with single interrupted sutures. This cannula is designed for long-term use in VAD patients and not a regular atrial cannula for cardiopulmonary bypass. We think that this is a way to effectively avoid thrombus formation in the LA.

Alternative bridge-to-lung transplant strategies in patients with PH are balloon atrioseptostomy (at our center preferably combined with the implantation of an atrial flow regulator) or the creation of a reverse Potts-shunt (either surgically or interventionally). Both procedures increase cardiac output and decrease RV afterload but carry the risk of cyanosis by right-to-left-shunting. Therefore, these procedures can only be applied in PH patients who are not oxygen dependent because the increase in cardiac output goes along with a decrease in oxygen saturation (17, 18).

In conclusion, long-term PLAD support presents a feasible therapeutic option for patients suffering from severe PH and RV failure as a bridge-to-lung transplantation. The patient can be fully awake and perform physical therapy much easier than with venoarterial ECMO, which puts him in a better condition for the following lung transplant. Nevertheless, daily inspections of the paracorporeal circuit for thrombotic depositions and liberal system exchanges are necessary to avoid thromboembolic complications even when cannulae are implanted that are designed for long-term use. Further studies are necessary to confirm our findings.

## Data Availability

The original contributions presented in the study are included in the article, further inquiries can be directed to the corresponding author.
